# Water Use Enhancement and Root Function Compensatory Regulation of Biomass Accumulation in Quinoa Under Salt Stress by Photosynthetic Drive Advantage

**DOI:** 10.3390/plants14111615

**Published:** 2025-05-25

**Authors:** Hao Xu, Lingzheng Feng, Jia Hao, Yongkun Zhang, Runjie Li

**Affiliations:** 1 School of Civil and Hydraulic Engineering, Qinghai University, Xining 810016, China; haoxu996@163.com; 2 Land Remediation and Ecological Restoration Center, Department of Natural Resources of Qinghai Province, Xining 810001, China; 13997142169@163.com (L.F.); haojiazrzy@163.com (J.H.); 3 State Key Laboratory of Plateau Ecology and Agriculture, Qinghai University, Xining 810016, China

**Keywords:** quinoa, water–salt stress, biomass, RF model, PLS-PM model

## Abstract

Water and salt stress significantly impact the accumulation of crop biomass (TB); however, the relative contributions of photosynthetic, physiological, and morphological factors remain poorly understood. This study aims to comprehensively investigate the effects of water and salt stress on crop growth physiology and identify the primary factors influencing biomass accumulation. We examined four quinoa varieties (*Qingli No.1, Qingli No.8, Gongza No.4, and Black quinoa*) under four salinity levels (s0: 0 mmol/L, s1: 100 mmol/L, s2: 200 mmol/L, and s3: 300 mmol/L) and two moisture levels (w1: 30% field capacity (FC), w2: 80% FC). Using principal component analysis (PCA) and correlation analysis, we constructed a random forest model (RF) and a partial least-squares path modeling (PLS-PM) framework to elucidate the effects of water and salt stress on quinoa growth physiology and clarify the adaptive mechanisms of quinoa under varying salinity conditions. The results indicate that (1) salinity has a more substantial regulatory effect on the accumulation of proline (Pro) and sodium ions (Na^+^) than water availability. Under conditions of adequate moisture (w2), the activity of antioxidant enzymes increased in response to mild salinity stress (s1). However, with escalating salinity levels, a significant decrease in enzyme activity was observed (*p* < 0.05). (2) PCA identified salinity as a key factor significantly influencing physiological changes in quinoa growth. The RF model indicated that, under severe salinity conditions (s3), intrinsic water-use efficiency (iWUE) emerged as a critical driver affecting biomass (TB) accumulation. (3) The PLS-PM model quantified the relative contribution rates of various factors to total biomass (TB). It revealed that, as salinity increased, the path coefficients of photosynthetic factors also rose, but their relative contribution diminished due to a corresponding reduction in the contribution of morphological factors. These findings offer a theoretical foundation and decision-making support for the integrated management of water–salt conditions in saline–alkali agricultural fields, as well as for the cultivation of salt-tolerant crops.

## 1. Introduction

Crop biomass refers to the total organic matter produced by a crop during a specific growth period. This biomass results from photosynthetic assimilation in the above-ground parts of the plant and nutrient uptake by the root system [1]. As a crucial indicator of crop growth and development, biomass reflects not only the accumulation patterns of dry matter within the plant but also the overall efficiency of energy conversion in crops. The capacity for biomass accumulation in crops is influenced by a combination of intrinsic genetic factors and external environmental conditions [2,3,4]. Genetically, interspecific differences among crops, such as variations in photosynthetic and water-use efficiencies, as well as physiological and metabolic characteristics among different cultivars of the same species, can significantly affect the rate of biomass accumulation [5,6]. Externally, both biotic and abiotic stresses can severely constrain normal biomass accumulation by impairing the functionality of photosynthetic organs and altering root morphology [1,7].

In the context of global climate change, drought and salinity stress have emerged as significant abiotic factors that limit biomass accumulation in crops [8,9,10]. These stressors primarily affect biomass accumulation by modulating intrinsic photosynthetic levels, physiological metabolic capabilities, and extrinsic morphological characteristics [7,9,11,12,13,14,15]. Regarding photosynthesis, stomata function as critical gateways for crops. Previous studies have shown that mild or short-term salt stress can induce physiological dehydration, leading to stomatal closure. This closure reduces stomatal conductance (Gs) and limits the net photosynthetic rate (A), adversely affecting biomass accumulation [16,17]. In contrast, severe or prolonged salt stress subjects crops to combined osmotic stress and ion toxicity, resulting in oxidative damage to photosynthetic mechanisms, further impacting biomass accumulation [17]. However, research on the response of biomass to crop photosynthesis under combined water–salt stress remains limited [7,18]. In terms of physiological mechanisms, existing research indicates that crops under water and salt stress regulate cellular osmotic pressure by increasing proline (Pro) content and enhancing the activity of antioxidant enzymes, such as catalase (CAT). These responses help mitigate the accumulation of reactive oxygen species (ROS) and maintain cellular viability, thereby influencing biomass accumulation [10]. Notably, salt stress primarily manifests through sodium ion toxicity; excessive sodium accumulation negatively affects crops’ osmotic potential, antioxidant capacity, and photosynthetic ability, consequently reducing biomass accumulation [19,20]. Morphological development is also significantly impacted by water and salt stress. The reduction in leaf area (LA) due to these stresses directly affects biomass accumulation, a finding that numerous studies have corroborated [21]. Key root morphological characteristics, such as total root length (L), root volume (V), and root surface area (SA), influence crops’ ability to acquire soil resources, thereby affecting biomass accumulation both directly and indirectly [22,23]. Additionally, root systems with similar mass but differing morphological traits can significantly alter the efficiency of resource acquisition from the soil [24]. Prior research has indicated that crops modify root architecture under abiotic stress to optimize resource acquisition strategies, which ultimately affects biomass production [1]. Although previous studies have explored the responses of biomass to photosynthesis, physiological mechanisms, and root morphology under combined water and salt stress [25], the relative contributions of these three processes to biomass accumulation remain inadequately understood. Specifically, further investigation is needed into the dynamic regulatory mechanisms by which photosynthesis, physiological responses, and root morphology influence crop biomass under increasing stress levels.

Quinoa has attracted international attention for its tolerance to drought and salt–alkali conditions, positioning it as a promising candidate for widespread cultivation [8,26,27,28]. The Qaidam Basin in Northwest China, sharing geographical similarities with quinoa’s region of origin, has seen extensive cultivation of this crop. However, the prevalent issues of salinization and drought stress in the area pose significant challenges to the sustainable development of the quinoa industry [29]. The seedling stage is particularly vulnerable to water and salt stress [30,31], during which combined water–salt stress notably affects the photosynthetic performance, physiological mechanisms, and root morphology of various quinoa varieties [6,32]. This study focuses on four widely cultivated quinoa varieties from the Qaidam Basin and aims to achieve the following objectives: (1) to investigate the effects of water and salt stress on quinoa photosynthesis, physiological mechanisms, and root morphology; (2) to explore how water–salt stress alters the pathways and driving factors that regulate biomass variation; and (3) to propose suitable water management strategies for quinoa cultivation in arid and saline–alkaline regions.

## 2. Results

### 2.1. Leaf Water Status and Sodium Accumulation

**Figure 1:**
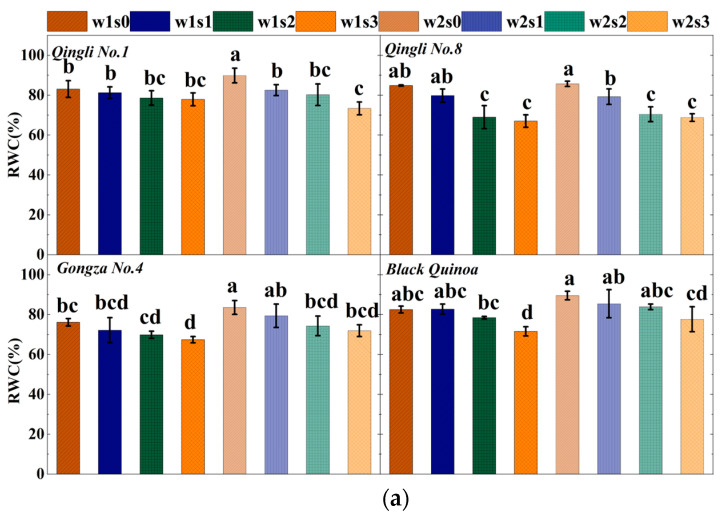

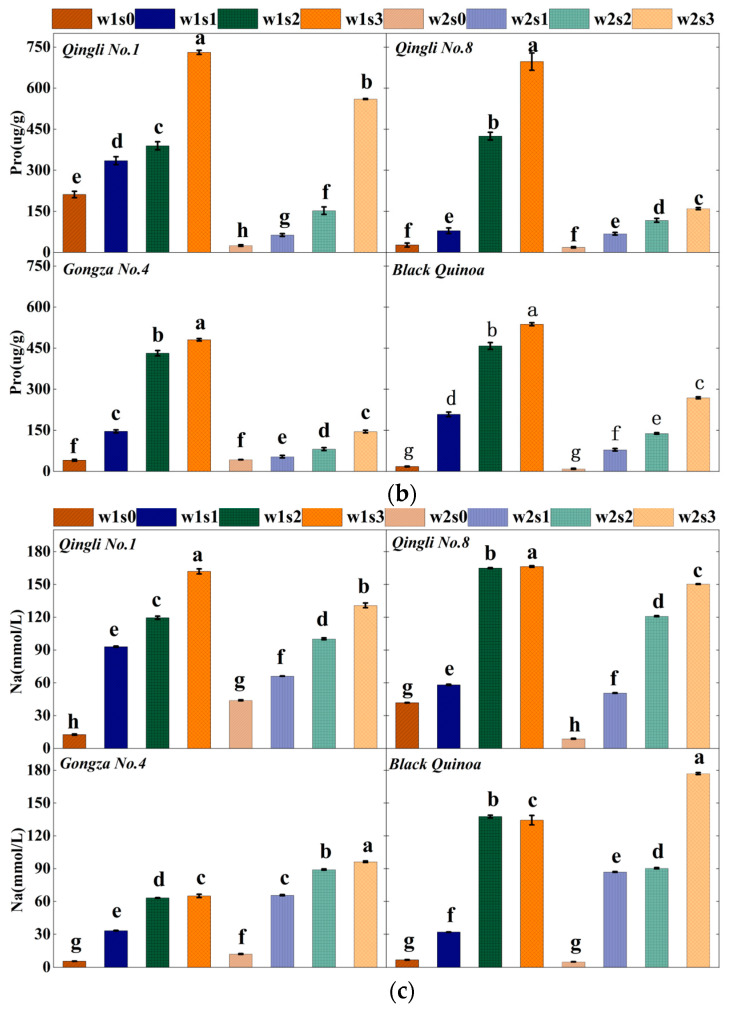
Water state of leaves of different varieties of quinoa under different treatments. (**a**) Relative water content of different treated leaves; (**b**) proline content in different treated leaves; (**c**) different treatment of sodium ion concentration in leaves. Note: Pro, proline; Na, sodium ion; RWC, relative water content. The different lowercase letters show significant differences at 0.05.

In response to progressively intensified water–salinity stress, the leaf water status of various quinoa cultivars exhibited consistent patterns (see Figure 1). The results indicate that water–salinity stress marginally reduced the relative water content (RWC) of leaves across the different quinoa cultivars. Under severe water–salinity stress (w1s3), RWC in the leaves of these cultivars significantly decreased by 13.30% to 21.77% compared to the non-stressed group (w2s0) (*p* < 0.05). In contrast, the decline in RWC for other treatments was generally not significant (*p* > 0.05). Under water–salinity stress, there was a significant increase in the accumulation of proline and sodium ions in the leaves of various quinoa cultivars. Specifically, compared to treatment w2, proline (Pro) accumulation increased by 162.89% (*p* < 0.05), while sodium ion (Na^+^) accumulation rose by 0.12% (*p* > 0.05) under treatment w1. Additionally, when compared to treatment s0, Pro accumulation significantly increased by 162.92%, 458.14%, and 810.93% (*p* < 0.05) with rising salinity gradients. Na^+^ accumulation also showed significant increases of 254.71%, 546.96%, and 689.99% (*p* < 0.05) under the same conditions. Overall, salinity had a more pronounced effect on the accumulation of proline and sodium ions in quinoa leaves. Furthermore, at equivalent salinity levels, adequate soil moisture significantly suppressed the accumulation of both proline and sodium ions.

### 2.2. Antioxidant Enzyme Activity and Photosynthetic Characteristics

**Figure 2:**
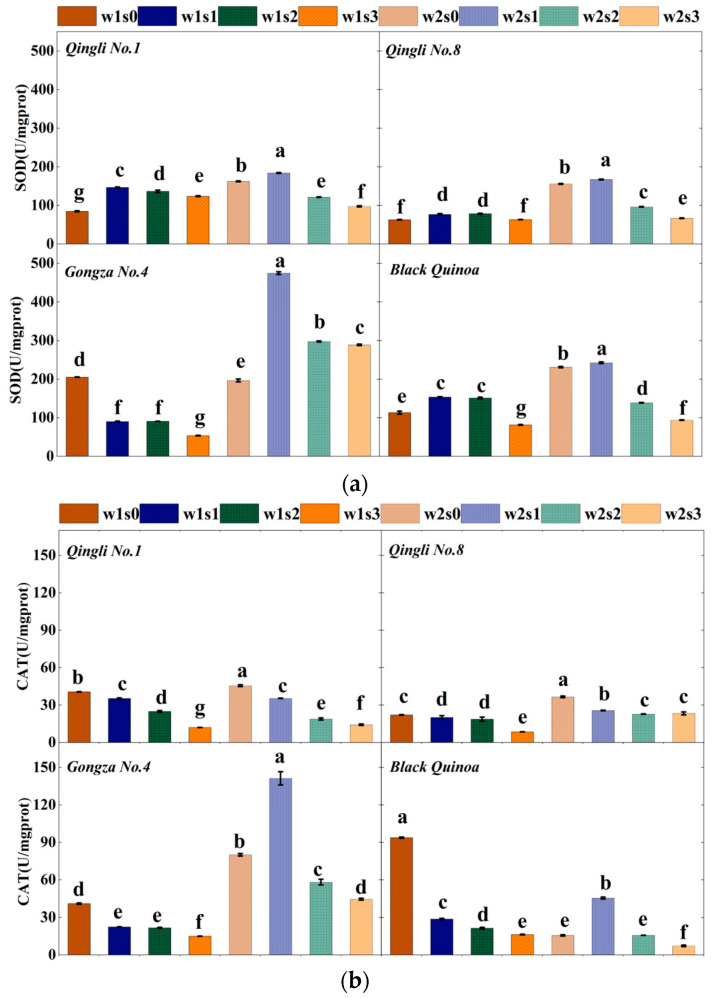

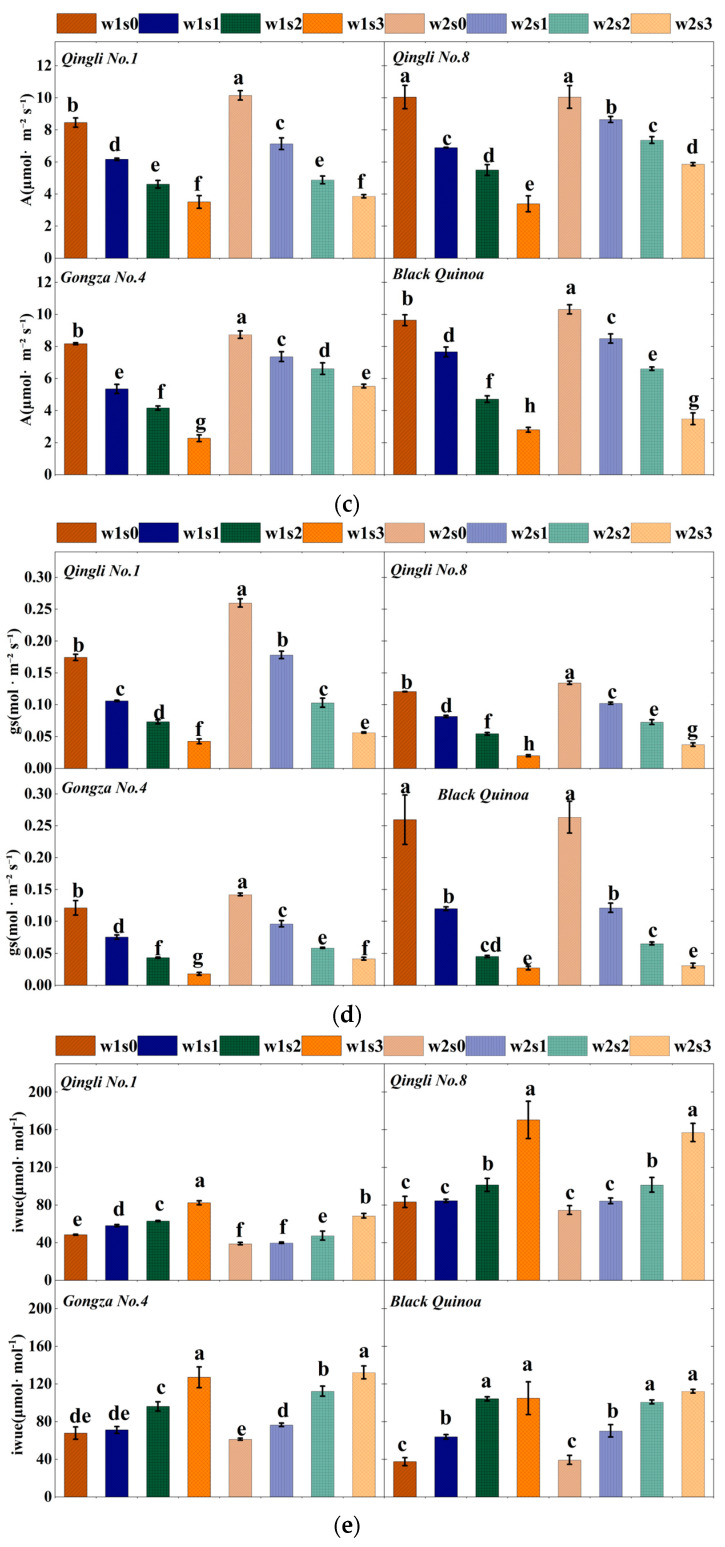
Activity and photosynthetic characteristics of antioxidant enzymes. (**a**) Different treatment effects on the activity of superoxide dismutase in leaves; (**b**) different treatment effects on the activity of peroxidase in leaves; (**c**) net photosynthetic rate of different treated leaves; (**d**) different treatment of leaf stomatal conductance; (**e**) different processing of intrinsic water-use efficiency within leaves. Note: A, net photosynthetic rate; Gs, stomatal conductance; iWUE, intrinsic water-use efficiency; SOD, superoxide dismutase; CAT, catalase. The different lowercase letters show significant differences at 0.05.

The response mechanisms of antioxidant enzyme activity to salinity vary slightly among different quinoa varieties, even under identical soil moisture conditions (Figure 2). Under well-watered conditions, *Gongza No. 4* shows enhanced antioxidant activity in response to salinity stress, with SOD and CAT activities significantly higher in the w2s1 treatment compared to other varieties and treatments (*p* < 0.05). Under treatment w1, leaf SOD and CAT activities decreased by 43.22% (*p* < 0.05) and 29.78% (*p* < 0.05), respectively. When comparing treatment s0 with other salinity gradients, SOD activity in each variety declined by −26.64%, 8.35%, and 28.29% (*p* < 0.05) as salinity increased. Similarly, CAT activity decreased by 5.58%, 46.10%, and 62.34% (*p* < 0.05) under the same conditions. Overall, the mean values of SOD (267.05 U/mg protein) and CAT (61.94 U/mg protein) were highest under the w2s1 treatment across all varieties. It can be inferred that under adequate moisture conditions (w2), mild salt stress enhances the activities of SOD and CAT; however, as salt stress increases, the activities of both enzymes decline.

The response patterns of stomatal conductance across various cultivars under water–salinity stress are similar to those observed in the net photosynthetic rate. Compared to treatment w1, the net photosynthetic rate (A) and stomatal conductance (Gs) decreased by 18.93% (*p* < 0.05) and 21.75% (*p* < 0.05) in the leaves, respectively. When compared to treatment s0, the net photosynthetic rate (A) decreased by 23.62%, 41.16%, and 59.35% (*p* < 0.05) as salinity increased. Correspondingly, Gs declined by 40.31%, 65.02%, and 81.43% (*p* < 0.05) with rising salinity. Overall, the net photosynthetic rate (A) of the various quinoa varieties significantly decreased with increasing salinity concentration. Additionally, while moisture significantly impacted the net photosynthetic rate, its effect was comparatively weaker than that of salinity. Under water–salt stress, all quinoa varieties showed an increase in intrinsic water-use efficiency (iWUE). Notably, *Qingli No. 8* exhibited a significantly higher iWUE at w1s3, measuring 170.44 μmol mol^−1^ (*p* < 0.05).

### 2.3. Morphological Characteristics and Total Biomass

**Figure 3:**
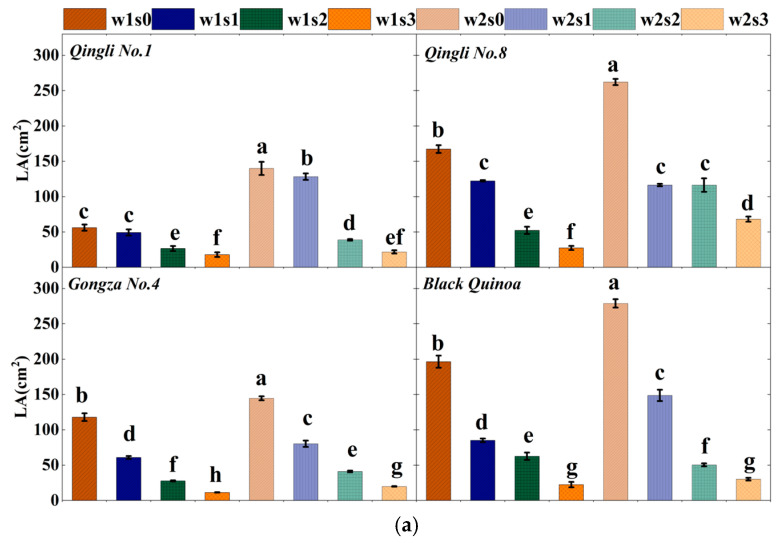

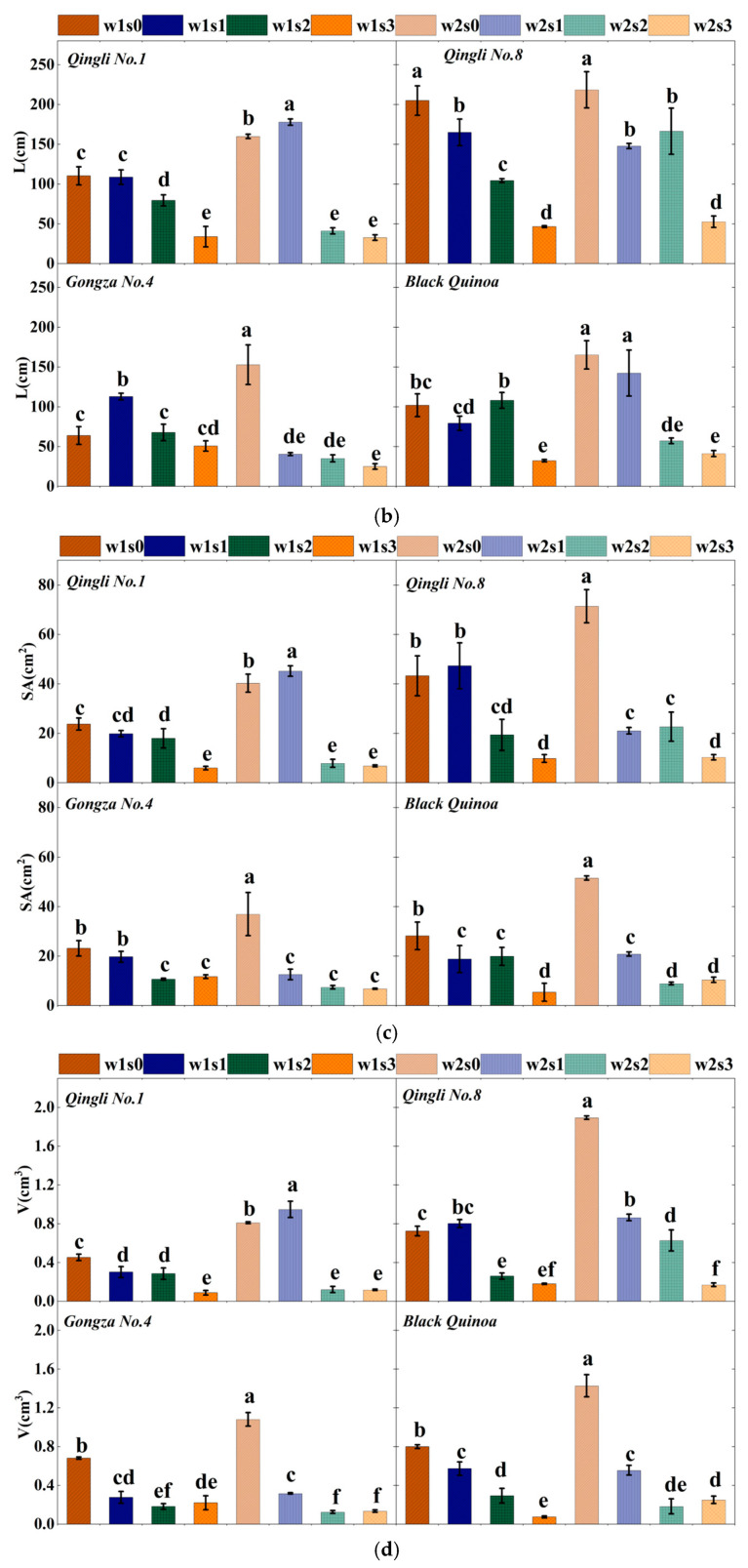

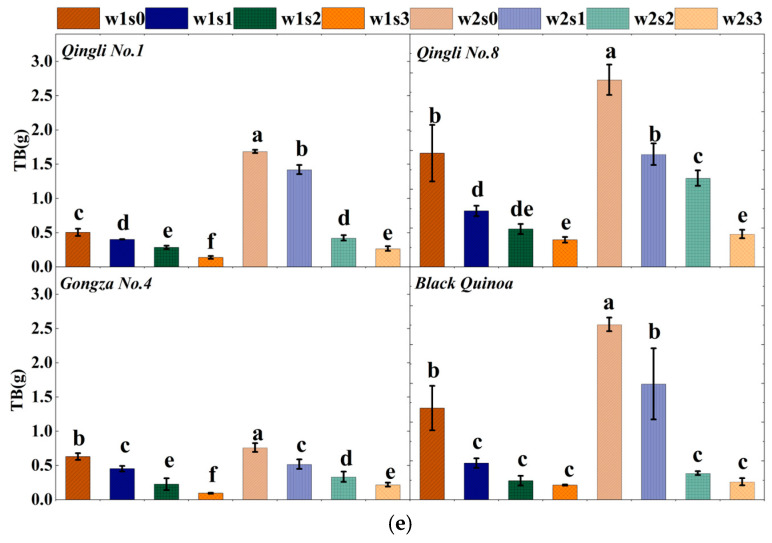
Morphological characteristics and total biomass of quinoa. (**a**) Different treatments of single leaf area; (**b**) different treatments of root length; (**c**) different treatments of root surface area; (**d**) different treatments of root system volume; (**e**) different treatments of single plant biomass. Note: LA, leaf area, SA, root surface area; L, root length; V, the root volume. The different lowercase letters show significant differences at 0.05.

Individual leaf area (LA) exhibited significant differences among various quinoa cultivars (Figure 3). As salt stress intensified, a marked reduction in LA was observed across all cultivars. Notably, the LA of *Qingli No. 8* and *Black quinoa* was significantly higher than that of *Qingli No. 1* and *Gongza No. 4*, highlighting phenotypic variations among cultivars in response to pre-salt stress conditions. In treatment w2, LA decreased by 34.54% (*p* < 0.05) compared to treatment w1. When comparing to treatment s0, LA decreased by 41.96%, 69.48%, and 83.92% for each variety, respectively, as the salinity gradient increased (*p* < 0.05). Under the same salinity gradient, a significant reduction in individual leaf area (LA) was observed in all varieties as soil moisture decreased (*p* < 0.05). Notably, *Qingli No. 1* demonstrated a particularly marked decrease in LA.

Significant differences exist in the response mechanisms of root length (L), root surface area (SA), and root volume (V) among various quinoa cultivars under water–salt stress. Compared to treatment w2, the metrics for L, SA, and V decreased by 11.27%, 14.81%, and 35.70%, respectively; however, these differences were not statistically significant (*p* > 0.05). When compared to treatment s0, root length (L) for each variety decreased by 17.26%, 43.98%, and 73.24% as salinity increased (*p* < 0.05). Similarly, SA decreased by 35.55%, 63.97%, and 78.89% (*p* < 0.05), while V decreased by 41.06%, 73.59%, and 84.26% with rising salinity. Overall, all root parameters significantly declined with the intensification of water–salt stress. Notably, under sufficient soil moisture conditions, *Qingli No. 1* exhibited significantly greater values for root length (L), root surface area (SA), and root volume (V) under mild salt stress (s1) compared to the unstressed control group (*p* < 0.05). Furthermore, total biomass for all cultivars showed a significant decreasing trend under increasing water–salt stress (*p* < 0.05), indicating a substantial impact of this stress on quinoa growth and development. Under similar water–salt stress conditions, *Qingli No. 8* consistently demonstrated higher biomass accumulation than other cultivars, suggesting its superior potential for biomass accumulation in response to water–salt stress.

### 2.4. Principal Component Analysis and Correlation Assessment

**Figure 4:**
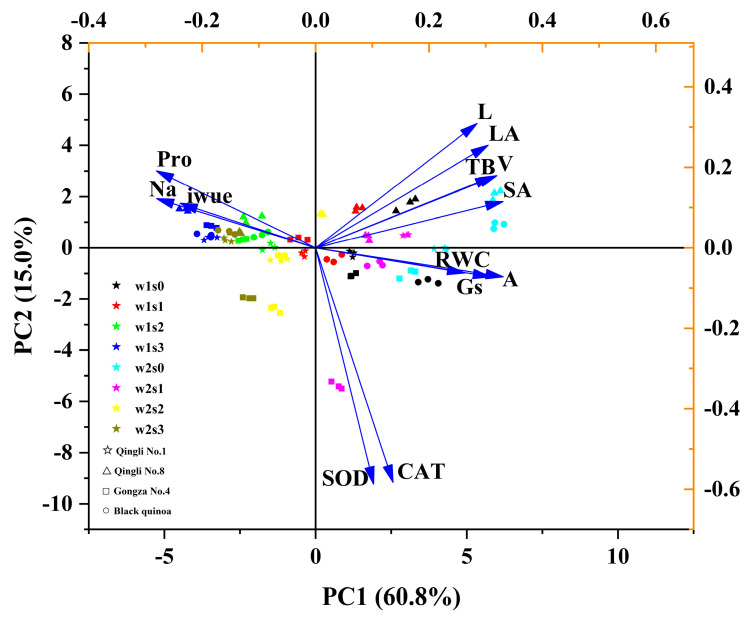
Principal component analysis. Note: A, net photosynthetic rate; Gs, stomatal conductance; iWUE, intrinsic water-use efficiency; Pro, proline; Na, sodium ion; RWC, relative water content; SOD, superoxide dismutase; CAT, catalase; LA, leaf area, SA, root surface area; L, root length; V, the root volume. Each punctuation color represents different water and salt treatment methods, and each punctuation shape represents different quinoa varieties.

The results of the principal component analysis (PCA) reveal that the first two principal components account for 75.8% of the total variance across all features. The first principal component (PC1) is particularly significant, accounting for 60.8% of the total variance (Figure 4). PC1 is primarily associated with the photosynthetic rate (A) and shows a notable positive correlation with relative water content (RWC) and stomatal conductance (Gs). The second principal component (PC2) primarily reflects root length (L) and exhibits a strong positive correlation with leaf area (LA), volume (V), total biomass (TB), and surface area (SA). This indicates a close relationship between PC2 and the morphological traits of quinoa. Moreover, the distribution of sample points along the PC1 axis shows no significant differences between varieties and moisture treatments. However, distinct differentiation is evident among the various salinity treatments (s0, s1, s2, and s3). This finding suggests that different salinity gradients may influence the interrelationships among quinoa traits.

**Figure 5:**
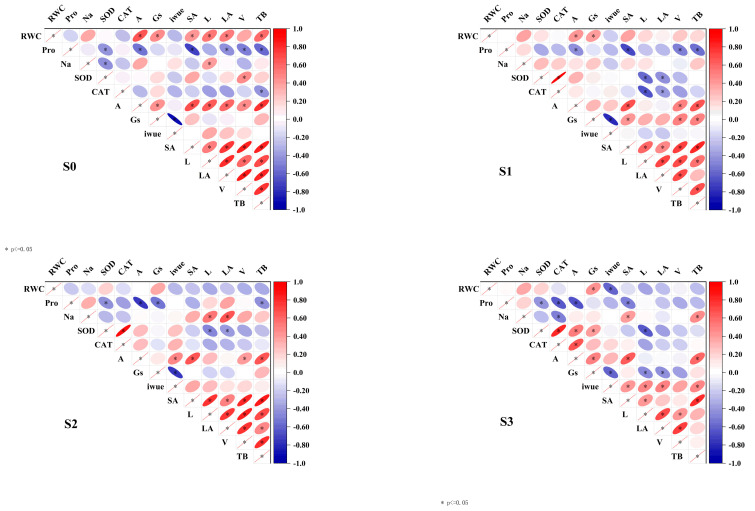
Correlation heat map. Note: A, net photosynthetic rate; Gs, stomatal conductance; iWUE, intrinsic water-use efficiency; Pro, proline; Na, sodium ion; RWC, relative water content; SOD, superoxide dismutase; CAT, catalase; LA, leaf area, SA, root surface area; L, root length; V, the root volume. * indicates significant difference in pairwise comparison. The red ellipse represents a positive correlation, and the blue ellipse represents a negative correlation. The ellipse represents the degree of correlation, and the narrower it is, the stronger the correlation is.

Analysis of the correlation heatmap reveals that total biomass (TB) consistently shows a significant positive correlation with photosynthetic rate (A) and root surface area (SA) across the increasing salinity gradient. As salinity increases, the correlation between total biomass (TB) and morphological traits (Figure 5)—such as root length, leaf area, and volume—gradually weakens. Additionally, no significant correlations were found between total biomass (TB) and stomatal conductance (Gs), intrinsic water-use efficiency (iWUE), or antioxidant enzyme activity. In the absence of salinity stress, proline (Pro) demonstrates a significant negative correlation with total biomass (TB), although this relationship weakens with increasing salinity levels.

Furthermore, the correlation between leaf water status indicators and photosynthetic parameters generally decreases as salinity rises. Regarding leaf antioxidant enzyme activity, there is no significant correlation between catalase (CAT) and superoxide dismutase (SOD) under the no-salinity treatment (s0). However, a significant positive correlation between the two is observed as salinity levels increase. In terms of morphological characteristics, strong positive correlations among the morphological indicators are evident at the s0, s1, and s2 stages. Conversely, at the s3 stage, these positive correlations significantly diminish. These findings provide valuable insights into the growth and physiological responses of quinoa’s total biomass under salt stress conditions.

### 2.5. Importance Analysis Based on Random Forest Model

**Figure 6:**
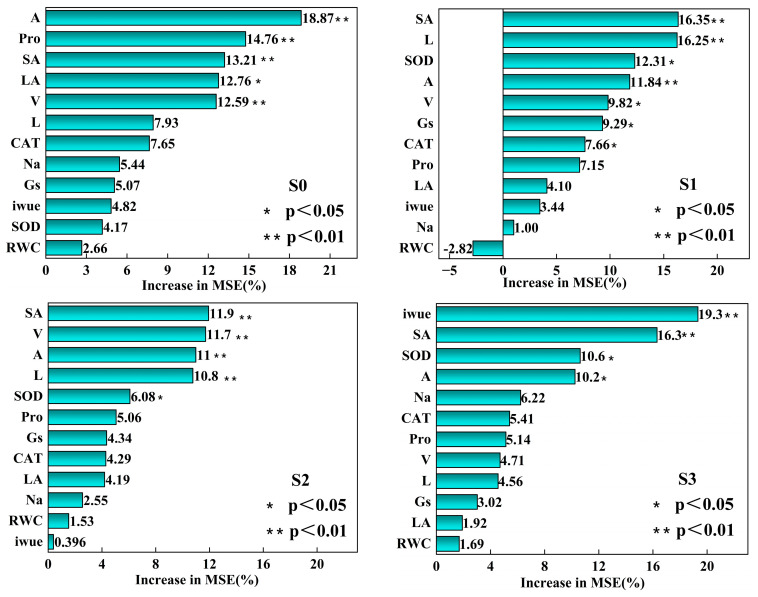
Relative importance of each indicator to total biomass. Note: A, net photosynthetic rate; Gs, stomatal conductance; iWUE, intrinsic water-use efficiency; Pro, proline; Na, sodium ion; RWC, relative water content; SOD, superoxide dismutase; CAT, catalase; LA, leaf area, SA, root surface area; L, root length; V, the root volume. The significance level of each predictor was * *p* < 0.05, ** *p* < 0.01.

This study investigates the primary factors that influence the total biomass (TB) of crops using random forest modeling to quantify the relative importance of each factor contributing to variations in total biomass. The Increased Mean Squared Error (IncMSE) serves as a comparative metric for assessing variable importance within predictive models, with higher values indicating greater significance for the variables. As shown in Figure 6, under salt-free conditions (s0), the net photosynthetic rate (A) is identified as the most significant driver of total biomass, with an IncMSE value of 18.87%. This value is significantly higher than the impacts of other variables (*p* < 0.01). Under mild salt stress (s1), root surface area (SA) and root length (L) emerge as the primary drivers of total biomass, while the influence of the net photosynthetic rate (A) diminishes. During moderate salt stress (s2), root-related parameters continue to play a central role in influencing total biomass; however, root volume (V) surpasses root length (L) in its impact. Under severe salt stress (s3), intrinsic water-use efficiency (iWUE) becomes the most significant driver affecting total biomass.

### 2.6. Analysis of Factors Driving Total Biomass in Quinoa

**Figure 7:**
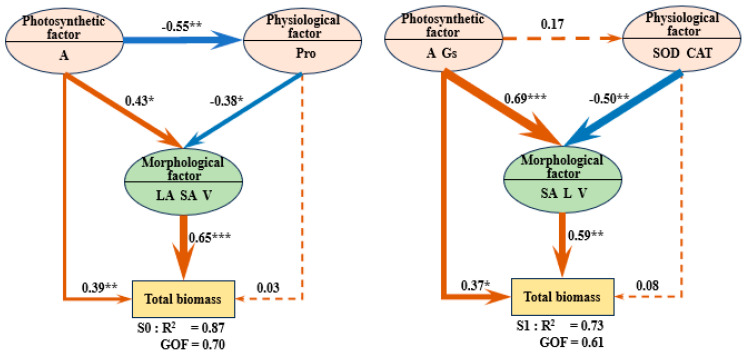

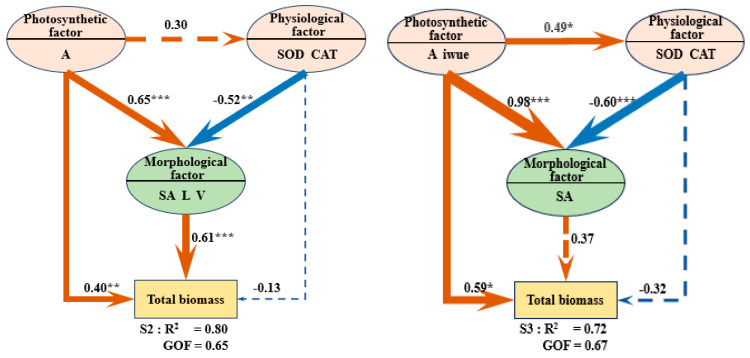
Structure of driving factors for total biomass. Note: The driving factor structure of total biomass of quinoa under different salt stress was constructed based on random forest model. It consists of the photosynthetic factor, physiological factor, morphological factor, and total biomass as outcome variables. A, net photosynthetic rate; Gs, stomatal conductance; iWUE, intrinsic water-use efficiency; Pro, proline; SOD, superoxide dismutase; CAT, catalase; LA, leaf area, SA, root surface area; L, root length; V, the root volume. A solid line indicates a significant relationship and a dashed line indicates a non-significant relationship. The thickness of the line indicates the strength of the causal relationship, supplemented by a standardized path coefficient. R2 represents the independent variable’s interpretation of the total change in the dependent variable, and GOF represents the goodness of fit for the entire model (GOF > 0.5). The significance level of each predictor was * *p* < 0.05, ** *p* < 0.01, *** *p* < 0.001.

A crop response model was developed using partial least-squares path modeling (PLS-PM) across four salinity gradients (Figure 7). This model quantifies the path coefficients of photosynthetic, physiological, and growth morphological factors influencing total biomass, elucidating the dynamic mechanisms behind their effects. Principal component analysis identified three latent variables, with photosynthetic factors (such as net photosynthetic rate and intrinsic water-use efficiency) and morphological factors (including leaf area and root architecture) playing pivotal roles in driving total biomass. As salinity stress intensifies, the positive impact of photosynthetic factors significantly increases, with the path coefficient rising from 0.39 to 0.59. In contrast, the contribution of morphological factors declines and loses statistical significance, as indicated by a decrease in the path coefficient from 0.65 to 0.37. Although physiological factors, specifically superoxide dismutase (SOD) and catalase (CAT), do not directly influence total biomass, their inhibitory effects on plant morphology intensify under increased salinity stress, as reflected in the path coefficient decreasing from −0.38 to −0.60. All goodness-of-fit (GOF) values for the model exceeded 0.6, indicating robust explanatory power.

Further analysis, combining random forest and correlation heatmap techniques, revealed that the gradient of salt stress significantly alters the composition of latent variable indicators. Photosynthetic factors transitioned from being predominantly governed by the net photosynthetic rate (A) to a synergistic interaction between A and intrinsic water-use efficiency (iWUE). Physiological responses shifted from being primarily driven by proline (Pro) to a combined antioxidant mechanism involving SOD and CAT. Growth morphological factors evolved from joint influences of leaf area (LA) and root parameters (SA, L, V) to reliance solely on root morphological adjustments. Under severe stress conditions (s3), the indicators of morphological factors became predominantly characterized by root surface area (SA). These results demonstrate that under salt stress, plants achieve adaptive regulation by adjusting photosynthetic strategies, optimizing antioxidant defenses, and reallocating resources to the root system. This provides quantitative evidence for understanding the growth and physiological mechanisms of plant responses to saline environments.

Table 1 presents the results of the PLS-PM model, detailing the impact of latent variables on the total biomass of quinoa. This includes direct effects, indirect effects, total effects, and relative contribution rates. Photosynthetic factors exert the most significant positive influence on quinoa’s total biomass, with a total effect exceeding 0.65. As the salinity gradient increases, the direct impact of photosynthetic factors rises; however, their relative contribution rate declines with increasing salinity. In contrast, physiological factors exhibit a negative overall impact on the variation in quinoa’s total biomass, with the absolute value of this negative effect increasing as the salinity gradient rises. This trend is accompanied by a gradual increase in their relative contribution rate, indicating that the inhibitory effects of physiological factors on biomass become more pronounced in high-salinity environments. Morphological factors, on the other hand, consistently show a positive overall influence, primarily reflected in their direct effects. However, as salinity increases, the relative contribution rate of morphological factors gradually decreases. This decline is particularly notable during the s3 phase, where a significant drop in the relative contribution of morphological factors is observed. This suggests that under high-salinity conditions, quinoa may struggle to maintain total biomass accumulation through morphological adjustments.

## 3. Discussion

### 3.1. Responses of Different Quinoa Varieties’ Growth Physiological Functions and Photosynthesis to Water and Salt Stress

As water and salt stress intensify, osmotic, ionic, and oxidative stresses become exacerbated. The absorption of harmful ions by the root system hinders cell division and disrupts metabolic processes. Consequently, crop photosynthesis and the root system’s ability to absorb water and nutrients are adversely affected, ultimately impairing crop growth and causing a slowdown or even cessation of biomass accumulation [33]. The reduction in relative water content (RWC) under abiotic stress allows crops to maintain cellular homeostasis by minimizing transpiration losses [34]. This study indicates that combined water–salt stress significantly decreased the RWC of quinoa leaves to 21.77% (*p* < 0.05). Similar results were reported by Mohammadi et al. [35], who found that drought and salinity stress markedly reduced the RWC of wheat leaves, supporting the conclusions of the present study. The accumulation of sodium ions (Na^+^) within the plant can significantly decrease osmotic potential and actively regulate osmotic balance [36]. However, prolonged exposure to water and salt stress can lead to sodium toxicity, resulting in cellular membrane damage. This condition subsequently affects various physiological activities, including osmotic regulation, photosynthesis, and metabolic processes, ultimately inhibiting biomass accumulation [37,38]. In this study, a significant accumulation of proline (Pro) was observed alongside an increase in Na+ levels. Similarly, research by Ami et al. [39] reported that both proline and Na+ concentrations significantly rose in wheat leaves under salt stress. This suggests that crops may utilize osmotic balance substances to manage stress. The synthesis of proline helps to alleviate the cellular osmotic pressure imbalance caused by the water deficit and may also offer indirect protection to membrane systems by scavenging reactive oxygen species (ROS), thereby reducing sodium ion toxicity [39]. Among the various cultivars studied, *Gongza No. 4* exhibited a more pronounced accumulation of proline under conditions of high salinity and low water availability, indicating its potentially superior capacity for osmotic regulation.

This study demonstrates that under adequate water conditions, mild salt stress generally enhances the activities of superoxide dismutase (SOD) and catalase (CAT) in cells. Similarly, Wang et al. [40] reported that drought and salt stress increase SOD and CAT activities in alfalfa cells. This enhancement is attributed to the excessive production of reactive oxygen species (ROS) induced by abiotic stress, which activates SOD and CAT—key antioxidant enzymes responsible for scavenging excess ROS and protecting cellular structures [40]. As the duration of drought and salt stress increases, the activities of superoxide dismutase (SOD) and catalase (CAT) significantly decline. Wang et al. [41] similarly reported a reduction in antioxidant enzyme activities in sorghum seedlings after 15 days of sustained drought and salt stress. This decline may result from the production of reactive oxygen species (ROS) outpacing the enzymes’ capacity to eliminate them, thereby disrupting the dynamic balance of antioxidants and impairing physiological metabolic processes. Consequently, this leads to an accumulation of oxidative stress within the cells. Additionally, elevated Na+ concentrations may compromise membrane integrity, inhibit enzyme stability, or suppress enzyme synthesis. This is evidenced by reductions of 28.29% and 62.34% in SOD and CAT activities, respectively, under salt stress conditions [42]. The correlation heatmap analysis revealed a significant negative correlation between the activities of superoxide dismutase (SOD) and catalase (CAT) and proline accumulation under severe salt stress (s3) in quinoa. Wang et al. [41] conducted a principal component analysis that demonstrated a close relationship between antioxidant enzyme activity and proline content under salt stress conditions, supporting the findings of the present study. This may indicate a resource reallocation strategy employed by quinoa in response to severe stress. When the dynamic balance of antioxidants is disrupted, proline (Pro) plays a crucial role in maintaining cellular viability through osmotic regulation and compensatory antioxidant mechanisms [41]. Additionally, *Gongza No. 4* demonstrated higher antioxidant enzyme activities under the w2s1 treatment (SOD: 267.05 U/mg protein; CAT: 61.94 U/mg protein), indicating a superior antioxidant stress response compared to other cultivars under mild salt stress. This characteristic holds potential application value for the improvement of mildly salinized agricultural fields.

Photosynthesis is the process by which light energy is converted into chemical energy, serving as the primary source of biomass and energy for plants. The results of this study indicate that as the severity of water–salt stress increases, both the net photosynthetic rate (A) and stomatal conductance (Gs) decrease concurrently. This finding is consistent with observations reported by other researchers [43,44,45]. We observed a significant decline in the net photosynthetic rate (A), primarily due to salt stress, with reductions of 59.35% in A and 81.43% in stomatal conductance (Gs) under the s3 treatment. The impact of the water deficit was less pronounced. This is because, under salt stress, plant cells experience not only osmotic effects but also unique influences such as sodium toxicity. Munns et al. [46] similarly found that salt stress inhibits the production of photosynthesis-related enzymes more severely than drought conditions. Furthermore, Ors et al. [47] reported that combined water and salt stress have a more significant negative impact on the net photosynthetic rate (A) and stomatal conductance (Gs) than either drought or salt stress alone, a conclusion that aligns with the findings of the present study. The simultaneous decline in stomatal conductance (Gs) and net photosynthetic rate (A) suggests that stomatal limitation is a crucial mechanism in the initial response to stress. However, under high salt stress, non-stomatal factors may gradually become more significant [48,49]. Intrinsic water-use efficiency (iWUE) is a vital physiological characteristic that emerges when plants are subjected to stress, as it reliably assesses how effectively crops conserve water under such conditions [50]. Among the evaluated varieties, *Qingli No. 8* maintained a relatively high iWUE of 170.44 μmol mol^−1^, even under severe stress. Apodaca et al. [50] similarly found that quinoa exhibits a significant increase in iWUE in response to combined water and salt stress. This suggests that quinoa can achieve a dynamic balance between stomatal and non-stomatal limitations by optimally regulating stomatal opening and closing [51]. This information may serve as a reference for breeding suitable varieties in arid regions. However, it is noteworthy that the enhancement of iWUE did not fully compensate for the loss in total biomass (TB), indicating that improvements in carbon fixation efficiency may come at the expense of growth rate.

Water–salt stress significantly inhibits the morphological characteristics of quinoa, with notable variations in leaf area (LA) and root traits observed among different varieties under such conditions. From a leaf phenotypic perspective, increasing water and salt stress led to a significant reduction in leaf area (LA) of various quinoa varieties, with a decrease of 83.92% compared to the unstressed group (*p* < 0.05). This reduction likely aims to minimize water loss by decreasing the surface area available for transpiration. However, it simultaneously compromises the plant’s ability to capture light. Similar findings have been reported by Ji et al. [52] and Alagoz et al. [53]. It is noteworthy that the leaf area of *Qingli No. 1* experienced a significantly greater reduction under water stress than under salt stress. Additionally, in the w2s1 treatment, the root length (L), surface area (SA), and volume (V) of *Qingli No. 1* increased by 17.26%, 35.55%, and 41.06%, respectively (*p* < 0.05). These results suggest that this variety’s carbon allocation strategy may prioritize the maintenance of root function [54]. This finding is consistent with the conclusions of Hussin et al. [6], who noted that certain quinoa varieties displayed enhanced root growth under mild salt stress. The differential responses of root morphology reveal deeper adaptive strategies. In crops experiencing soil water–salt stress, root organs act as primary sensing sites, playing a critical role in the overall response to these environmental challenges. Research indicates significant differences in the response mechanisms of root length (L), surface area (SA), and volume (V) among various quinoa varieties under water–salt stress. A common trend is that as the severity of water–salt stress increases, all root parameters tend to decline. The overall decline in total biomass (TB) supports the notion that water and salt stress disrupt biomass allocation networks on a global scale. This finding aligns with the results reported by Shannon et al. [55] and Aggarwal et al. [30], who observed similar effects in their studies on other crops.

### 3.2. Enhanced Water-Use Efficiency and Compensatory Root Function Regulation of Biomass Accumulation in Quinoa Driven by Photosynthetic Dominance Under Salt Stress

The combined analysis of principal component analysis (PCA) and correlation heatmaps indicates that salt stress drives the transformation of adaptive strategies in quinoa by reshaping the physiological and morphological association network. Principal component analysis (PCA) revealed a strong association between the first principal component (PC1) and photosynthetic parameters, particularly the net photosynthesis rate (A) and stomatal conductance (Gs), which together explained 60.8% of the variance. This relationship is further supported by the significant positive correlation between A and total biomass (TB) observed in the heatmap (*p* < 0.05), highlighting the crucial role of photosynthetic capacity in biomass accumulation under water and salt stress. Hussin et al. [56] suggested that optimizing photosynthetic efficiency mechanisms is essential for enhancing salt tolerance in forage species, which is consistent with the findings of this study. Furthermore, the strong association between TB and morphological parameters—including leaf area (LA), surface area (SA), length (L), and volume (V)—noted in the second principal component (PC2) emphasizes how biomass accumulation is responsive to changes in crop morphology. Similarly, Deok et al. [57] identified a significant correlation between alterations in root morphology and biomass in rice under stress conditions. The continuous distribution of samples from S0 to S3 along the PC1 axis highlights the significant impact of varying salt concentrations on crop physiological growth. Furthermore, the lack of clustering among the varieties supports the conclusions drawn by Hao et al. [58]. Moreover, the correlation heatmap indicates enhanced synergistic activity of superoxide dismutase (SOD) and catalase (CAT) under salt stress, reflecting the oxidative stress response mechanism triggered by salinity. This finding provides valuable data to support the physiological inhibitory effects observed in subsequent structural equation modeling analyses.

The variable importance ranking derived from the random forest model quantitatively characterizes the three-stage adaptive trajectory of quinoa: “photosynthetic dominance”, “root remodeling”, and “water conservation”. This trajectory reveals the plant’s shift from a strategy prioritizing carbon acquisition to one focused on water conservation [58]. At the s0 stage, the net photosynthetic rate (A) demonstrates a significant advantage, with an IncMSE of 18.87%. This finding is supported by a strong correlation between A and total biomass (TB) observed in the correlation heatmap (*p* < 0.05). As salinity increased to S1, the importance of root parameters—specifically surface area (SA) and length (L)—rose sharply, accounting for 15.2% and 13.8% of the variance, respectively. This enhancement correlates with the increased association between SA and total biomass (TB) observed in the heatmap, indicating that crops may mitigate ion toxicity by optimizing root morphological structures. These findings are consistent with those reported by Zhou et al. [59]. By the s3 stage, intrinsic water-use efficiency (iWUE) becomes the primary factor influencing the adaptive strategy, with an IncMSE of 16.5%. This shift, along with the significant negative correlation between iWUE and stomatal conductance (Gs) observed in the heatmap (*p* < 0.05), indicates that quinoa adopts a survival strategy under salt stress by sacrificing some photosynthetic capacity to enhance the water-use efficiency. This finding is consistent with the adaptive regulation patterns identified in halophytes by Nuran et al. [5].

The PLS-PM model elucidates the hierarchical regulatory network governing the growth and physiological responses of quinoa under salt stress. Both photosynthetic and morphological factors positively influence biomass; however, the impact of photosynthetic factors is more pronounced, with the path coefficient increasing from 0.39 to 0.59 (*p* < 0.05). As salt stress intensifies, the relative contribution of photosynthetic factors gradually declines. This pattern arises primarily because, while the driving effect of photosynthetic factors on morphological factors has increased, the influence of morphological factors on biomass has gradually diminished. Specifically, the path coefficient at the s3 stage dropped to 0.37 (*p* > 0.05), leading to a decreased relative contribution of photosynthetic factors. This observation aligns with resource regulation patterns noted in stressed plants by Lin et al. [60]. Moreover, as salinity increases, the relative contribution of morphological factors also progressively decreases, particularly during the s3 stage where a significant decline is evident. This may indicate that under high-salinity conditions, quinoa struggles to maintain total biomass accumulation through morphological adjustments, highlighting the constraints imposed by salt stress on the plant’s morphological adaptability. While physiological factors, such as superoxide dismutase (SOD) and catalase (CAT), do not directly influence total biomass, they indirectly affect biomass accumulation by negatively regulating morphological parameters. The path coefficients for these relationships decrease from −0.38 to −0.60 (*p* < 0.05). This suggests that activating the antioxidant defense system under salt stress requires substantial resource allocation, which may limit morphological plasticity. This phenomenon of “metabolic costs of stress resistance” parallels the carbon allocation trade-offs observed in salt-tolerant plants, as described by Tyerman et al. [61], providing a quantitative framework for understanding the growth-defense balance in saline environments. All models exhibited a goodness of fit (GOF) greater than 0.6 and an R^2^ value exceeding 0.7, demonstrating strong explanatory power across varying salinity stress gradients. This establishes a theoretical framework for managing different saline–alkaline agricultural fields.

### 3.3. Moderated Irrigation Management to Address the Impact of Intrinsic Drivers on Biomass in Quinoa Under Varying Salinity Stress

This study elucidates the dynamic regulation of biomass accumulation in quinoa under combined water and salinity stress through various mechanisms. Principal component analysis indicates that the salinity gradient is a crucial variable, reshaping the interactive network of photosynthetic, physiological, and morphological factors that govern biomass accumulation patterns in quinoa. In the PLS-PM model, the relative contributions of photosynthetic factors range from 47.59% to 38.37%, highlighting their predominant role in biomass formation. This finding underscores the synergistic effects of the net photosynthetic rate (A) and stomatal conductance (Gs) in driving biomass accumulation [61]. As salinity levels increase from s1 to s2, the ratio of contributions from photosynthetic to morphological factors diminishes. Coupled with random forest (RF) analysis, it becomes evident that root surface area (SA) and root length density (L) surpass photosynthetic parameters, emerging as key drivers of biomass accumulation. This suggests that quinoa optimizes its survival strategy through adjustments in root morphology, evidenced by an increase in the incremental mean square error (IncMSE) of SA by 32.6 ± 4.8%. Under high-salinity conditions (s3), the relative contribution of morphological factors declines significantly, while the IncMSE of intrinsic water-use efficiency (iWUE) increases to 22.3%. Nieves et al. [62] indicated that when plants cannot adapt morphologically to elevated salinity levels, they may enhance carbon assimilation per unit of water to maintain survival. This study quantitatively analyzes this outcome using RF and PLS-PM models, revealing a “damage control logic” in resource allocation for crops in saline–alkaline environments.

Despite the high water deficit in the experimental setup (30% CF), principal component analysis (PC1 variance contribution of 60.8%) indicates that salinity is the primary limiting factor influencing physiological photosynthetic responses and biomass accumulation in quinoa. This finding highlights the importance of assessing soil salinity levels in target areas before quinoa cultivation. Effective soil salinity management can help ensure that quinoa employs a “carbon acquisition prioritization” strategy during its early survival stages, rather than being forced to switch to a “water conservation prioritization” mode. In addition, irrigation strategies in water-scarce regions can be adjusted to improve crop water-use efficiency while managing soil salinity levels. Cocozza et al. [63] demonstrated that during the early stages of quinoa cultivation, plants improve their drought and salt–alkali resistance through root morphological plasticity in response to mild water or salinity stress, without significantly affecting yields. This observation aligns with the adaptive responses observed in *Qingli No. 1* under mild salinity stress. In the PLS-PM model, the contribution of physiological factors was found to increase significantly with rising salinity levels. This finding suggests that salt stress can markedly inhibit biomass accumulation by modulating crop stress resistance metabolism; however, sufficient water availability can mitigate this effect. Overall, these findings underscore quinoa’s potential as a promising alternative crop for cultivation in water-scarce regions or mildly saline–alkaline soils. Nonetheless, given the limited water gradients in this study, further research is needed to assess the extent of soil moisture retention under mild salinity stress that does not significantly inhibit biomass accumulation.

## 4. Materials and Methods

### 4.1. Experimental Site and Design

**Figure 8:**
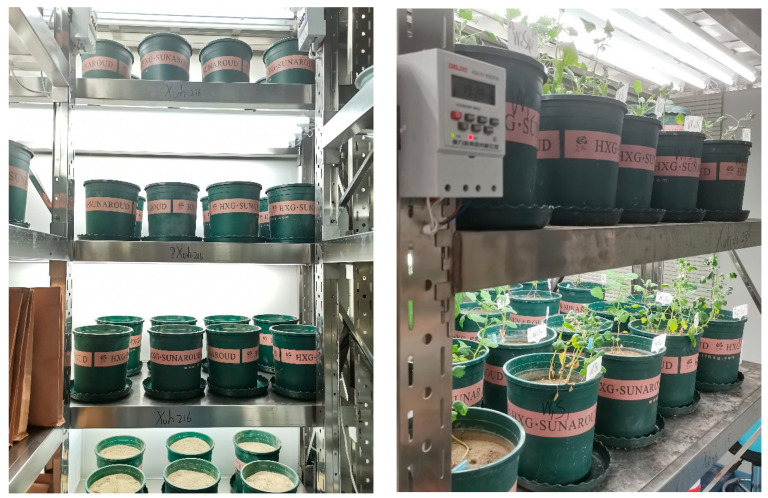
Potted plant experiment scenario.

The experiment was conducted in a controlled greenhouse environment at the State Key Laboratory of Ecological Agriculture and Plateau Pastoralism at Qinghai University in Xining City, Qinghai Province, China. Throughout the study, external conditions were carefully regulated. Plants were grown under a 14 h photoperiod from sowing until the end of the experiment. Average diurnal temperatures were maintained at 24 °C during the day and 20 °C at night, with relative humidity levels set at 70% during the day and 80% at night. The photosynthetic photon flux density was established at 400 μmol m^−2^ s^−1^, provided by a combination of warm-white, fluorescent lamps [32]. To minimize the effects of environmental gradients within the greenhouse, plants were randomly repositioned weekly (Figure 8). The quinoa varieties used in this experiment are listed in Table 2, with seeds sourced from the College of Animal Science and Technology at Qinghai University in Xining City, Qinghai Province, China. All four varieties are high-quality, high-yield cultivars commonly cultivated in the region.

Soil samples were collected from the Qaidam Basin, situated in the northeastern and northwestern regions of the Tibetan Plateau in China (87°49′ E to 99°19′ E, 34°41′ N to 39°20′ N). This basin is characterized by a plateau continental climate, featuring dry, cold winters and arid, windy summers. The growing season extends from May to October, with an annual average temperature of 4.5 °C and a mean temperature of 12 °C during this period. In the central and western parts of the basin, the average evaporation rate is approximately 2600 mm, about 50 times the annual average precipitation. In contrast, the southern and eastern regions exhibit an annual average evaporation rate of 1900 mm, which is six times the annual average precipitation. The annual average wind speed exceeds 2.5 m s^−1^ [64].

The soil is classified as yellow loam, characterized by a loamy texture. Total nitrogen, total phosphorus, available nitrogen, and available phosphorus were measured using a Continuous Flow Analyzer (CFA, SEAL AA500, SEAL Analytical GmbH, Werkstraße 5, 22844 Norderstedt, Germany) [65]. Soil pH was determined with a pH meter (INES-3C, Shanghai, China) using the electrode method at a soil-to-water ratio of 2.5:1 [66]. Total salt content was assessed using the gravimetric method [67], while soil field capacity was measured using the ring knife method [68]. The results indicated total nitrogen of 1.7 g/kg, total phosphorus of 3.08 g/kg, available nitrogen of 29.89 mg/kg, and available phosphorus of 4.68 mg/kg. The average field capacity (FC) was 15.83%, the soil pH was 7.81, and the total salt content was 1.33 g/kg, classifying it as mildly saline–alkali soil. All soil samples were air-dried and passed through a 2 mm sieve before being layered into containers with a controlled bulk density of 1.45 g/cm^3^. To ensure uniformity in the filling process, the soil was layered in increments of 5 cm.

Irrigation levels were established at full irrigation (80% field capacity, FC) and deficit irrigation (30% FC) [9,43]. Research indicates that a salt concentration of 300 mmol/L significantly affects quinoa traits [32,69]. Accordingly, four salinity gradients were developed based on this standard. The irrigation water was prepared to match the local saline composition, with a mass ratio of NaCl to CaCl_2_ to Na_2_SO_4_ to NaHCO_3_ approximately 1:5.35:3.58:9.27. Each treatment was replicated three times, as detailed in Table 3. Quinoa seeds are available in various colors, including white, black, red, brown, and gray. However, the primary varieties used in production are white, black, and red. This study does not focus on plant breeding terminology; thus, the classification of quinoa varieties follows the nomenclature established by Chen et al. [70]. Additionally, in accordance with local agricultural practices, a side dressing of fertilizer is applied one month after quinoa emergence, with each pot receiving ten granules of compound fertilizer.

The experiment employed a pot cultivation method using standardized planting pots. Each pot contained five quinoa plants of similar morphology and size, and each treatment included three pots. After a natural growth period of five weeks post-germination, varying levels of water–salt stress were applied for an additional five weeks, corresponding with the initial signs of wilting in the plants. Data collection occurred after the stress treatment concluded. All treatments were exposed to identical light conditions, and the positions of the planting pots were periodically rotated to minimize edge effects.

### 4.2. Gas Exchange Parameters

Following the study conducted by Ma et al. [71], the Li-6800 open gas exchange system was utilized to measure gas exchange parameters in the mature upper leaves of quinoa. The analysis focused on the fully expanded third mature leaf from the top of the quinoa plant. Gas exchange parameters were measured using the Li-6800 system (Li-Cor, Lincoln, NE, USA).

The methodology for measuring gas exchange rates in quinoa leaves was outlined by Jon et al. [32]. Assessments began three hours after the greenhouse lights were turned on, maintaining a temperature of 24 °C and relative humidity at 60%. The photosynthetic photon flux density was set at 400 μmol m^−2^ s^−1^, with a CO_2_ concentration of 400 μmol·mol^−1^. The net photosynthetic rate (A) and stomatal conductance (Gs) were calculated using Li-Cor software (http://www.licor.com). The intrinsic water-use efficiency (iWUE) was determined by dividing A by Gs.

### 4.3. Antioxidant Enzyme Activity and Sodium Accumulation

Approximately 0.5 g of fresh leaf samples from three different plants were ground and homogenized, then filtered through fine mesh cloth. The resulting homogenate was centrifuged at 10,000× *g* for 10 min at 4 °C [72]. The supernatant was used to measure the activities of catalase (CAT) and superoxide dismutase (SOD) in the leaf cells. CAT activity was assessed by measuring the rate of hydrogen peroxide decomposition, as indicated by absorbance at 240 nm, following the method described by Bauza et al. [73]. SOD activity was evaluated using the WST-1 method [69], employing a detection kit from Nanjing Jiancheng Bioengineering Institute.

All root, stem, and leaf samples were subjected to a kill-green treatment by heating at 105 °C. The samples were then dried at 80 °C until a constant weight was attained, after which they were ground for analysis. Following the method outlined by Hussin et al. [56], a quantified amount of the processed samples was digested using a mixture of HNO_3_ and HClO_4_. The concentration of sodium ions was determined using an atomic absorption spectrophotometer, with the results reported in mmol/L.

### 4.4. Leaf Water Parameters

After photographing the leaf area, we selected leaves randomly for immediate weighing to obtain the fresh weight (FW). The leaves were submerged in deionized water for 12 h, then gently blotted dry and reweighed to determine the turgid weight (TW). Subsequently, the leaves were placed in an oven at 70 °C until they reached a constant weight to obtain the dry weight (DW). The relative water content (RWC) was calculated using the formula described by Neil et al. [74]: RWC (%) = [ (FW − DW)/(TW − DW) ] × 100.

The proline (Pro) concentration was determined using the method outlined by Bates et al. [75]. A leaf tissue sample (20 mg, freeze-dried) was homogenized in 2 mL of 3% sulfosalicylic acid and centrifuged at 16,100 g for 5 min. The supernatant was then stored on ice. To 0.75 mL of the supernatant, 0.75 mL of ninhydrin reagent was added. This reagent was prepared by dissolving 1.25 g of ninhydrin in 20 mL of 6 M phosphoric acid and 30 mL of glacial acetic acid. Next, an additional 0.75 mL of glacial acetic acid was added to the mixture of supernatant and ninhydrin reagent. The samples were incubated at 100 °C for 1 h. After cooling the tubes, 1.5 mL of toluene was added, and the mixture was vigorously shaken for 20 s. The resulting fluid separated into two phases, with the upper phase collected for analysis. The absorbance was then measured at 517 nm. The proline content was expressed as μg g^−1^ fresh weight (FW).

### 4.5. Plant Morphological Characteristics and Biomass

Harvested plants were divided into leaf, stem, and root sections, and their fresh weight (FW) was recorded. The samples were then dried at 80 °C until a constant weight was achieved, allowing for the measurement of dry weight (DW). The leaves were photographed for documentation, and leaf area (LA) was calculated using image analysis software. At the end of the experiment, root systems were harvested by randomly selecting three representative plants from each treatment. The roots were carefully excavated and rinsed with distilled water to remove any excess soil and substrate debris.

After removing excess moisture from the roots with absorbent paper, the root systems were scanned using an HP LaserJet Pro M226 MFP scanner. The images were analyzed with Win RHIZO Pro root analysis software (http://agripheno.com) to obtain key parameters, including total root length (L), total root surface area (SA), and total root volume (V).

### 4.6. Data Processing

Data were organized using Excel 2019. Spearman correlation analysis, random forest (RF) modeling, and partial least-squares path modeling (PLS-PM) were employed to examine the relationships among growth morphology, physiological characteristics, and photosynthetic traits of quinoa. These methods also aimed to identify the primary drivers influencing total biomass. Correlation and significance analyses were conducted using IBM SPSS Statistics version 26.0. The RF model and PLS-PM were performed using R version 4.3.3, while graphical representations of the results were generated with Origin 2022 software.

## 5. Conclusions

(1) Quinoa total biomass (TB) exhibits a comprehensive response to combined water and salinity stress, influencing photosynthetic activity, physiological mechanisms, and morphological adaptation strategies. Increased salt concentration led to a significant decrease in the relative water content (RWC) of the leaves (*p* < 0.05). In contrast, maintaining an optimal soil moisture level of 80% field capacity effectively reduced the excessive accumulation of proline (Pro) and sodium ions (Na^+^), thereby alleviating the cellular damage caused by salt stress. The photosynthetic system exhibits a high sensitivity to salt stress, as demonstrated by a significant reduction in the net photosynthetic rate (A) and stomatal conductance (Gs) under the s3 treatment (*p* < 0.05). In contrast, the intrinsic water-use efficiency (iWUE) showed a notable increase in Qingli No. 8 (*p* < 0.05). These findings suggest that under severe salt stress, quinoa significantly improves its water management efficiency to adapt to critical survival conditions. Morphologically, leaf area (LA) and root parameters, including length, surface area, and volume, generally exhibited a decreasing trend with increasing water–salt stress. However, Qingli No. 1 showed an unusual increase in root metrics under the w2s1 treatment, underscoring the inter-varietal differences in growth strategies in response to stress conditions.

(2) The combined analysis using the random forest (RF) model and partial least-squares path modeling (PLS-PM) elucidated the dynamic reconfiguration patterns of quinoa’s growth adaptation strategies under water–salinity stress. The RF model indicated that, as salinity increased, the primary driver affecting biomass shifted from the net photosynthetic rate (A, IncMSE = 18.87%) to intrinsic water-use efficiency (iWUE, IncMSE = 19.3%). This shift reflects an adaptive change in photosynthetic strategy, moving from a focus on biomass accumulation to prioritizing crop survival under stress conditions. The path coefficient analysis using the PLS-PM model indicated that the path coefficients of photosynthetic factors increased with the salinity gradient (*p* < 0.05). However, the relative contribution rate gradually decreased, primarily due to a reduction in the contribution of morphological factors. This decline was particularly pronounced under the s3 treatment, suggesting that quinoa faces challenges in maintaining biomass accumulation through morphological adjustments under high-salt-stress conditions. Simultaneously, the increasing relative contribution of physiological factors with rising salinity gradients suggests an intensifying inhibitory effect on morphological development, thereby impacting biomass accumulation. These findings provide a theoretical basis for developing planting strategies that consider adaptive transitions in growth physiological mechanisms under varying salinity conditions, as well as for dynamic water and salt regulation strategies in saline–alkaline farmland.

## Figures and Tables

**Table 1:** The influence of different factors on biomass.

Salinity Level	Latent Variable	Direct Impact	Indirect Influence	Overall Impact	Contribution (%)
s0	Photosynthetic factor	0.39	0.4	0.79	47.59
	Physiological factor	0.03	−0.25	−0.22	13.25
	Morphological factor	0.65	N.A.	0.65	39.16
s1	Photosynthetic factor	0.37	0.37	0.74	47.74
	Physiological factor	0.08	−0.3	−0.22	14.19
s2	Morphological factor	0.59	N.A.	0.59	38.06
	Photosynthetic factor	0.4	0.26	0.66	38.37
	Physiological factor	−0.13	−0.32	−0.45	26.16
	Morphological factor	0.61	N.A.	0.61	35.47
s3	Photosynthetic factor	0.59	0.1	0.69	43.13
	Physiological factor	−0.32	−0.22	−0.54	33.75

Note: The direct influence is the path coefficient between two variables, the indirect influence is the sum of the products of the relevant path coefficients, the total influence is the sum of the direct and indirect influences, and the relative contribution rate is the absolute value of the total effect as a proportion.

**Table 2:** Different quinoa varieties.

Seed Color	Crop Variety
White	*Qingli No.1*
White	*Qingli No.8*
Red	*Gongza No.4*
Black	*Black quinoa*

**Table 3:** Different water- and salt-stress treatments.

Treatment Group	Irrigation Level	Salinity Level
w1s0	30% FC	0 mmol/L
w1s1	30% FC	100 mmol/L
w1s2	30% FC	200 mmol/L
w1s3	30% FC	300 mmol/L
w2s0	80% FC	0 mmol/L
w2s1	80% FC	100 mmol/L
w2s2	80% FC	200 mmol/L
w2s3	80% FC	300 mmol/L

## Data Availability

The data presented in this study are available on request from the corresponding author.
